# Parasite and Pesticide Impacts on the Bumblebee (*Bombus terrestris*) Haemolymph Proteome

**DOI:** 10.3390/ijms24065384

**Published:** 2023-03-11

**Authors:** Dalel Askri, Edward A. Straw, Karim Arafah, Sébastien N. Voisin, Michel Bocquet, Mark J. F. Brown, Philippe Bulet

**Affiliations:** 1 Plateforme BioPark d’Archamps, 74160 Archamps, France; 2 Centre for Ecology, Evolution & Behaviour, Department of Biological Sciences, School for Life Sciences and the Environment, Royal Holloway University of London, Egham TW20 0EX, UK; 3 Department of Botany, School of Natural Sciences, Trinity College Dublin, D02 PN40 Dublin, Ireland; 4 Phylogene S.A. 62 RN113, 30620 Bernis, France; 5 Apimedia BP22-Pringy, 74371 Annecy, France; 6 CR, University Grenoble Alpes, IAB Inserm 1209, CNRS UMR5309, 38000 Grenoble, France

**Keywords:** Amistar, *Crithidia bombi*, differential proteomics, glyphosate, immune response, MALDI BeeTyping, sulfoxaflor

## Abstract

Pesticides pose a potential threat to bee health, especially in combination with other stressors, such as parasites. However, pesticide risk assessment tests pesticides in isolation from other stresses, i.e., on otherwise healthy bees. Through molecular analysis, the specific impacts of a pesticide or its interaction with another stressor can be elucidated. Molecular mass profiling by MALDI BeeTyping^®^ was used on bee haemolymph to explore the signature of pesticidal and parasitic stressor impacts. This approach was complemented by bottom-up proteomics to investigate the modulation of the haemoproteome. We tested acute oral doses of three pesticides—glyphosate, Amistar and sulfoxaflor—on the bumblebee *Bombus terrestris*, alongside the gut parasite *Crithidia bombi*. We found no impact of any pesticide on parasite intensity and no impact of sulfoxaflor or glyphosate on survival or weight change. Amistar caused weight loss and 19–41% mortality. Haemoproteome analysis showed various protein dysregulations. The major pathways dysregulated were those involved in insect defences and immune responses, with Amistar having the strongest impact on these dysregulated pathways. Our results show that even when no response can be seen at a whole organism level, MALDI BeeTyping^®^ can detect effects. Mass spectrometry analysis of bee haemolymph provides a pertinent tool to evaluate stressor impacts on bee health, even at the level of individuals.

## 1. Introduction

Pollinators are vital components of natural and managed ecosystems, contributing USD 235–577 billion a year to the global economy through the ecosystem service of pollination [1]. The majority of this economically important pollination is carried out by bees [1], but numerous studies indicate that bees are threatened and in decline [2,3], posing a threat to pollination services. A range of anthropogenic pressures are believed to threaten bee health, from land-use change [4] to pesticides [5,6,7], acting both individually and in combination [8]. Two stressors in particular—pesticides and parasites—have received significant research attention due to the large impacts they may have on bee health [5,7,9,10,11,12,13,14,15,16]. The impact of these stressors can be measured at a range of levels, from populations to molecules [15,17,18,19]. Studies at the molecular level can provide an understanding of the mechanistic interaction between stressors and bee health [15,20]. As such, they underpin and explain studies of lethal and sublethal effects in individuals, colonies, and populations [10,11,13,14,15,21,22]. In addition, changes in gene expression or protein production in response to stressors may provide potential biomarkers that can be used in health monitoring [11,12,17,23,24,25,26,27].

Previous molecular-level studies in bees exposed to pesticides and parasites have largely focused on gene expression. For example, Haas et al. (2022) analysed genomic data for 75 bee species and demonstrated by the recombinant expression of 26 CYP9Q3 putative functional orthologs that detoxification is an evolutionarily conserved mechanism across bee families [28]. They observed a conserved capacity to metabolise certain insecticides across all major bee families while identifying a limited number of bee species where this function may have been lost. Similarly, Al-Naggar and Baer (2019) studied the effects of short-term exposure to a sublethal dose of the flupyradifurone-based insecticide Sivanto early in life on survival and immunity in *A. mellifera* [12]. They selected five genes involved in detoxification, CYP305D1, CYP6AS14, CYP9Q3, GSTD, and SODH2 as representatives of antioxidant-enzyme families that are known to target pesticides and secondary metabolites [29,30], along with genes involved in the insect immune response. The *defensin1* gene was down-regulated compared to controls, while the *apismin*, *Lys-1*, and *chitinase* genes were significantly up-regulated in pesticide-exposed bees compared to control bees. Glyphosate exposure can alter immune response pathways by down-regulating the gene expression coding for host-produced AMPs (abaecin, apidaecin, defensin, and hymenoptaecin) in *A. mellifera* [27]. Moreover, *A. cerana cerana* and *A. mellifera ligustica* are affected by glyphosate commercial formulation [31]. However, this exposure seems to increase rather than decrease the expression of many genes involved in immunity, agrochemical detoxification and resistance, such as antimicrobial peptides, cuticle proteins, and cytochrome P450 families. In other studies, immune/detoxifying gene expression was variable (up and down) [23,25]. No significant pesticide–parasite interactions were found for any of the genes investigated. Proteomic changes in bees exposed to parasites and/or pesticides are also an area of active research. By analysing haemolymph proteome, several physiological functions of honey bees, such as energy metabolism, detoxification, metamorphosis, and chemosensing, have been shown to be disrupted by *Varroa* [32]. Up-regulation of proteins involved in stress response, carbohydrate metabolism and energy synthesis, and protein folding/binding was observed in the head proteome of nurse honey bees [33]. Houdelet et al. (2021) found changes to the gut proteome following exposure of *A. mellifera* to *Nosema* spp. [22]. The team observed both up- and down-regulation of various proteins mainly involved in metabolism and response to stimuli. Previously, fipronil was also observed to induce important neuroproteomic changes in the brains of honey bees [34]. 

However, the majority of these studies have used the managed honey bee *A. mellifera* [35,36,37], which is not representative of the more than 20,000 species of bee [38], and have focused on insecticides rather than other types of pesticide [35,36,37]. Here we investigate proteomic responses of the bumble bee *Bombus terrestris*, an abundant and important wild and managed pollinator of crops and wildflowers [1,39,40], to individual and combined exposure to a range of agrochemicals and the highly prevalent parasite *Crithidia bombi* [41,42,43,44]. To address the broad range of agrochemicals, we use (i) sulfoxaflor, a sulfoximine insecticide that has been shown to detrimentally affect bumble bee health [45,46,47] and that was banned in the EU in 2022 for outside use (EC, 2022); and (ii) Amistar, a broad-spectrum fungicide product containing azoxystrobin, which saw high uptake in the early 2000s and is still used widely today. Amistar is the flagship formulation for the active ingredient azoxystrobin, although it has now moved out of patent, and 71 other azoxystrobin products are available in the UK alone (Straw and Stanley, In Review). An emulsifier/surfactant co-formulant in Amistar, alcohol ethoxylates, has been found to cause damage to bumblebee gut tissue, leading to food aversion, weight loss, and ultimately death [48]. Finally, (iii) glyphosate, a herbicide that is the world’s most used pesticide [49,50]. The impacts of glyphosate on bees are hotly contested [51,52], with the most rigorous evidence pointing to potential sublethal impacts on the microbiome [53], with conflicting evidence as to lethal effects [54,55,56]. To represent the most likely co-exposure situation in the field, we used the trypanosome gut parasite *C. bombi*, which is the most prevalent parasite of bumble bees across Europe [41,42,43,44]. *C. bombi* impacts physiology [57,58], learning [59,60,61], and colony fitness [58,62]. 

Using modified OECD risk assessment protocols and fully crossed experiments, combined with MALDI BeeTyping^®^ and bottom-up proteomics by LC-ESI-MS/MS [22,63,64], we ask (i) how exposure to an insecticide, fungicide, and herbicide, individually or in combination with the parasite, impacts the haemolymph proteome profile; (ii) which proteins respond to these stressors; and (iii) how these responses map onto higher level effects of exposure on individual longevity, weight change, and parasite load.

## 2. Results and Discussion

### 2.1. Whole-Organism Metrics

#### 2.1.1. Survival

In the Amistar experiment, Amistar caused significant mortality (41.4%), as did the Amistar + *C. bombi* treatment (18.8%). No bees in the negative control or *C. bombi*-only treatments died, while all bees in the positive control died, confirming the test’s ability to detect lethal effects. The global chi-square test found a significant impact of treatment (X^2^ (2, *n* = 92) = 16.32, *p* < 0.001). Individually, the mortality impacts of Amistar and Amistar + *C. bombi* were significantly higher relative to the control (X^2^ (1, *n* = 49) = 13.55, *p* < 0.001) and (X^2^ (1, *n* = 63) = 4.43, *p* = 0.035). In the glyphosate and sulfoxaflor experiments, glyphosate did not cause any mortality to the bees, while sulfoxaflor exposure caused limited, but non-significant, mortality (5.6%), similar to the combined sulfoxaflor + *C. bombi* exposure (9.1%). No bees in the negative control, *C. bombi* only, glyphosate only or glyphosate + *C. bombi* treatments died, while all bees in the positive control (dimethoate) died, confirming the test’s ability to detect lethal effects. Due to low mortality, Fisher’s Exact tests were exclusively used for a treatment versus control comparison. There was no significant effect of either sulfoxaflor alone or the sulfoxaflor + *C. bombi* treatment on mortality relative to the control (Fisher’s exact test (two-sided) *p* = 0.190 and *p* = 0.053, respectively).

#### 2.1.2. Weight Change 

In the Amistar experiment, bees in the positive control gained the most weight, while all other treatments gained less weight, or even lost weight. Bees in the positive control on average gained 20.9 mg, while bees in the *C. bombi* treatment gained 11.9 mg on average. Bees exposed to Amistar alone gained less weight, at just 6.2 mg, while Amistar + *C. bombi*-exposed bees lost an average of 4.8 mg. The weight change in the *C. bombi* alone treatment was not significantly different to the control (PE = −0.01, CI = −0.02 to 0.0). In contrast, the weight changes in the Amistar-only and Amistar + *C. bombi* treatments were significantly different relative to the control (Amistar only: PE = −0.02, CI = −0.03 to −0.0; Amistar + *C. bombi*: PE = −0.03, CI = −0.04 to −0.01). In the glyphosate and sulfoxaflor experiments, bees in the positive control lost some weight, while all other treatments made limited weight gains. Bees in the positive control on average lost −2.5 mg, while bees in the *C. bombi* treatment gained 8.6 mg on average. Glyphosate-only bees gained 1.9 mg, while glyphosate + *C. bombi*-exposed bees gained 0.5 mg. Sulfoxaflor-only bees gained the most weight at 11.3 mg, while sulfoxaflor + *C. bombi*-exposed bees gained 6.5 mg. However, relative to the control, no weight loss or gain was statistically significant (*C. bombi*: PE = 0.01, CI = −0.00 to 0.03; glyphosate only: PE = 0.00, CI = −0.01 to 0.02; glyphosate + *C. bombi*: PE = 0.0, CI = 0.01 to 0.02; sulfoxaflor only: PE = 0.01, CI = −0.00 to 0.03; sulfoxaflor + *C. bombi*: PE = 0.01, CI = −0.00 to 0.02).

For the whole organism metrics, impacts varied by substance. Amistar caused significant mortality and weight loss (or lack of weight gain). This mirrors the effects found in Straw and Brown (2021) [48], which found that a co-formulant, alcohol ethoxylates, was responsible for these effects, while the active ingredient (Azoxystrobin) did not contribute to the mortality effects. The weight loss in the Amistar + *C. bombi* treatment, and significant lack of weight gain in the Amistar-only treatment was likely caused by melanisation of the gut tissue, reducing appetite and a bee’s ability to intake energy. Ultimately, this likely explains the mortality seen in these treatments. The reduced mortality in the Amistar + *C. bombi* treatment is likely stochastic, as there is little reason *C. bombi* would ameliorate the impacts of the pesticide. It is worth noting that this work pre-dates the experiments in Straw and Brown (2021) [52], so the mortality was unexpected, hence the sample size for the haemoproteome analysis is reduced as only living bees had haemolymph extracted.

Neither glyphosate nor sulfoxaflor, nor their combination with *C. bombi*, caused any significant impacts on survival or change in weight. These findings confirm prior findings that acute exposure to glyphosate has little to no measured impact on these metrics [52]. Sulfoxaflor caused a non-significant amount of mortality, although 5–9% indicates that the 0.06 µg dose used was potentially beyond our intention of a fully non-lethal dose. That no impacts were noted with this high exposure gives confidence that sulfoxaflor does not impact these traits. 

No pesticides caused an impact on parasite intensity, indicating that they do not meaningfully interact over this timescale or with this exposure. While this experimental design is more parametrised to detect pesticidal effects, the lack of change in parasite intensity suggests that even with an experiment tailored to detect parasite-driven effects, none would be seen. For all whole organism metrics, there was no impact of *C. bombi*, even alongside pesticide exposure. This reaffirms prior results showing that in OECD 247 style acute toxicity tests, *C. bombi* does not contribute to mortality [52]. Additionally, it confirms previous findings, using different methods, that *C. bombi* does not meaningfully interact with pesticidal stressors [47,52].

### 2.2. Molecular Mass Fingerprints (MFPs) 

**Figure 1:**
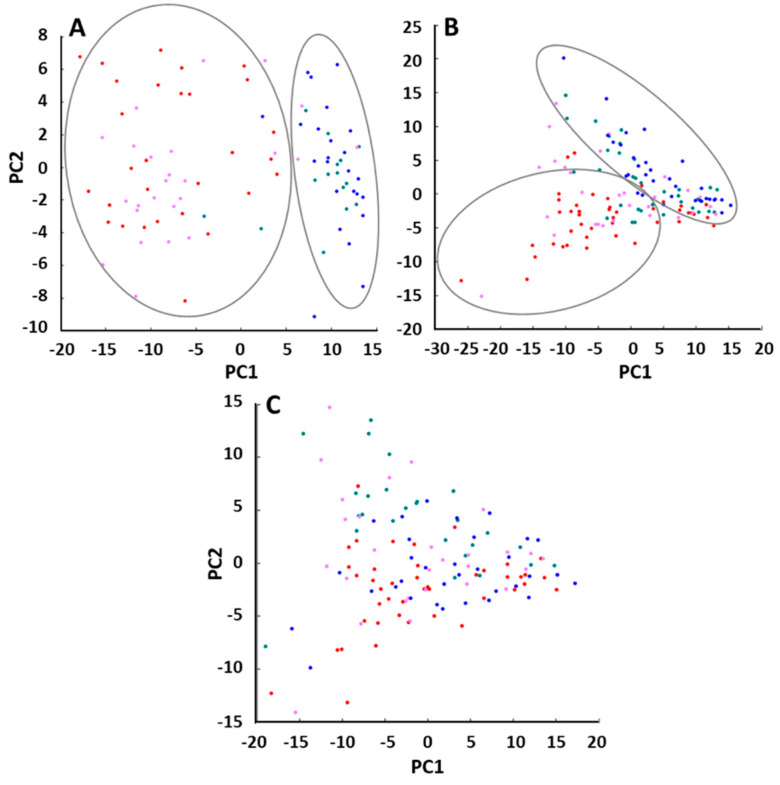
Principal component analyses (PCA) to discriminate molecular effects on *Bombus terrestris* following Amistar (**A**), glyphosate (**B**), and sulfoxaflor (**C**) exposure. Pesticide alone or in combination with *C. bombi.* Red: control, green: pesticide, pink: *C. bombi*, blue: pesticide + *C. bombi*. Each point represents the haemolymph molecular mass fingerprints from an individual bee. PCAs are generated from ClinProTools™.

Exposure to Amistar, either alone or in combination with the parasite *C. bombi*, impacted the haemoproteome when compared to control bees or bees exclusively infected with *C. bombi*, (Figure S1). No discrimination between *C. bombi* parasitised and control bees, nor Amistar treated versus Amistar infected with *C. bombi*, was observed. By comparing the PCAs of the control, Amistar, *C. bombi*, and Amistar + *C. bombi* experiments, there was a clear separation between the two groups exposed to Amistar versus the control and *C. bombi* (Figure 1A). 

A similar separation was observed in the PCAs of control versus glyphosate, control versus glyphosate + *C. bombi* and glyphosate versus glyphosate + *C. bombi* (Figure S2). In the case of sulfoxaflor, based on the PCAs, bees treated with sulfoxaflor alone were discriminated from the control bees and from bees infected with *C. bombi* alone. In our experimental conditions, an infection with *C. bombi* did not lead to discrimination between samples (Figures S2 and S3). This is concordant with what we observed in the pairwise analysis. For glyphosate, there was a tentative differentiation between glyphosate and glyphosate + *C. bombi* versus *C. bombi* and the control (Figure 1B). For sulfoxaflor, there was no discrimination as all groups overlapped (Figure 1C).

### 2.3. Modulated Molecular Ions (MMIs) Following Amistar, Sulfoxaflor, and Glyphosate Exposure and Co-Infection with C. bombi

**Figure 2:**
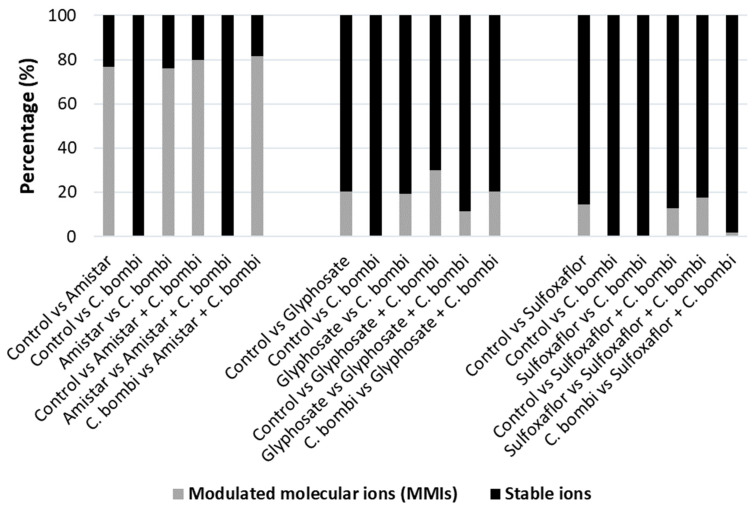
Percentage of the modulated molecular ions (MMIs) that discriminate molecular mass fingerprints of *B. terrestris* following exposure to Amistar, glyphosate, and sulfoxaflor. The graph was generated based on ClinProTools™ peak lists for each pairwise comparison. All ions with *p* < 0.0083 (0.05/6) from a Wilcoxon–Kruskal–Wallis test were considered as significant.

Supporting the PCA data, a high number of significantly modulated molecular ions (MMIs) were detected in the Amistar experiment (Amistar-exposed bees versus (i) control (76.92% MMIs), (ii) *C. bombi* alone (76.24%), (iii) *C. bombi* with Amistar (79.90%), and (iv) when we compared *C. bombi* to Amistar + *C. bombi*-treated bees (81.55%)). Lower numbers of significant MMIs were observed in the glyphosate and sulfoxaflor experiments, as shown in Figure 2. Across all three experiments, we did not observe any significant MMIs following *C. bombi* infection alone. The details of total, stable and modulated ions for all pairwise comparisons are available in Table S1.

### 2.4. Variation in Three Bee Immune Peptides—Apidaecin, Abaecin, and Chymotrypsin Inhibitor—Following Pesticide Exposure

To understand the differences in molecular ion levels, we analysed the generated peak lists and focused on peptides that are recognised as indicators of an activated bee immune response (namely apidaecin, abaecin, and chymotrypsin inhibitor) with average molecular-related ions identified by MALDI BeeTyping^®^ as m/z 1978.6, 4396.5 and 5937.8, respectively. Apidaecin and abaecin peak values responded similarly across treatments, but chymotrypsin inhibitor responded differently (Table 1). The details of the percentage calculation are available in Table S2. Chymotrypsin inhibitor was reported to be impacted by bee stressors such as the *Nosema* parasite in *A. mellifera* [22], and could be a bee health response marker in *B. terrestris*.

Under glyphosate and sulfoxaflor exposure, the average molecular-related ions of apidaecin and chymotrypsin inhibitor did not change (*p* > 0.05) (Table S2). Furthermore, no significant variation was noted for abaecin in any of the treatments. However, apidaecin varied significantly following Amistar exposure. Statistical analysis showed that only Amistar exposure led to significant changes in apidaecin (PWKW control versus Amistar < 0.000001, Amistar versus *C. bombi* 0.0000013, control versus Amistar + *C. bombi*, and *C. bombi versus* Amistar + *C. bombi* < 0.000001) and chymotrypsin inhibitor (PWKW control versus Amistar 0.000387, Amistar versus *C. bombi* 0.000407, control versus Amistar + *C. bombi*, and *C. bombi* versus Amistar + *C. bombi* < 0.000001). 

### 2.5. Protein Quantity Variations Following Pesticide and Parasite Exposure Demonstrated by Differential Bottom-Up Proteomics

**Figure 3:**
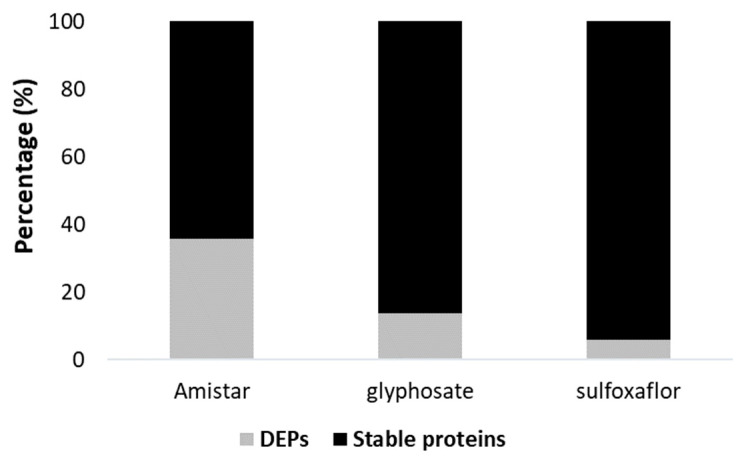
Percentage of proteome changes following exposure to Amistar, sulfoxaflor, and glyphosate. Differentially expressed proteins (DEPs). Proteins with a ratio < 0.5 (down-regulation) and >2 (up-regulation) with *p* < 0.05.

Using LFQ, we were able to quantify a total of 621 proteins, including 369 unique proteins, across the experiments (Table S3). Among them, 65 unique proteins were differentially expressed (DEPs), reflecting an impact on the proteomes by a given experimental treatment (Table S4). The results of this section are reported by experiment, i.e., all the different treatment groups related to a pesticide. Interestingly, the highest percentage of DEPs was observed after Amistar exposure (35.69%), followed by glyphosate (13.81%) and sulfoxaflor (5.95%) (Figure 3).

**Figure 4:**
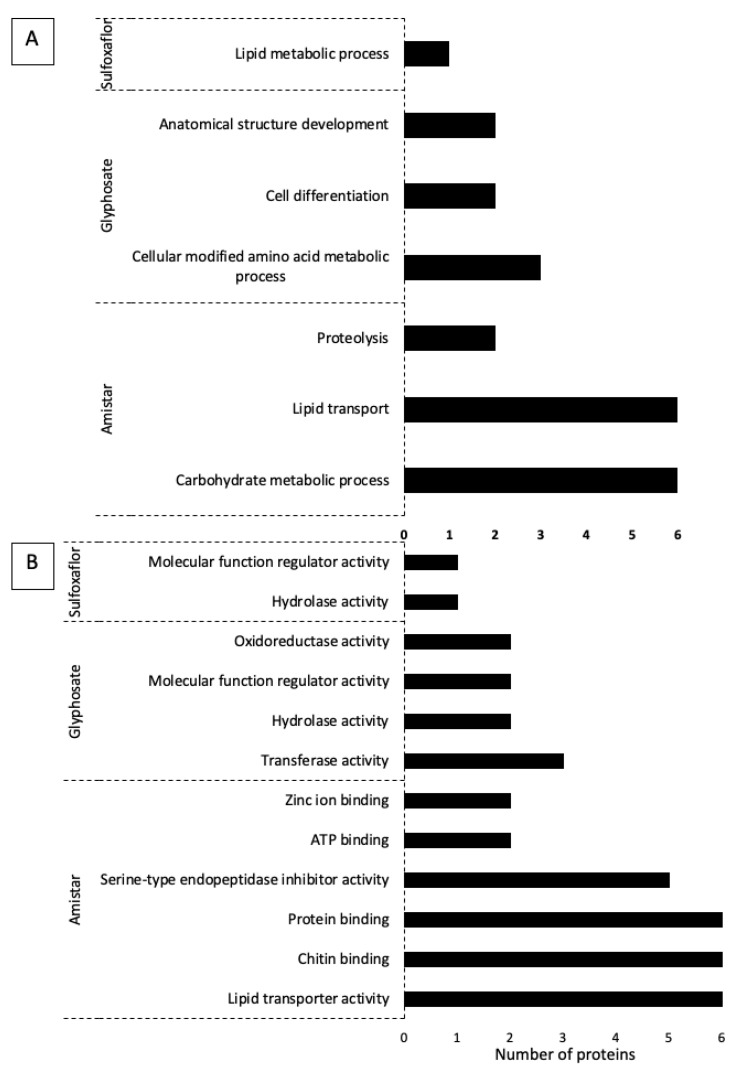
Functional distribution of the dysregulated proteins following Amistar, glyphosate, and sulfoxaflor exposure according to the most impacted (**A**) biological processes and (**B**) molecular functions. Assignments were made with Blast2Go tool within OmicsBox. The number indicates the number of proteins.

The proteins that were dysregulated following *C. bombi* exposure compared to any of the other conditions were not parasite-specific, as they were also seen in the remaining comparisons. This is in concordance with the lack of an effect of the parasite on the whole-body metrics. For further analysis and interpretation, we focused on the DEPs and analysed their variation per pesticide, i.e., Amistar, glyphosate, and sulfoxaflor. A Venn diagram (Figure S4) was generated to identify proteins detected only in a specific treatment or proteins that were DEPs across the different exposures. Of the 65 dysregulated proteins, 46 unique proteins were found after Amistar exposure, 13 after glyphosate, and 4 after sulfoxaflor. Two proteins were differentially expressed under two pesticide treatments: peptidoglycan recognition protein SA, ATL64812.1 after Amistar or glyphosate exposure, and uncharacterised protein LOC107189219 (XP_015433190.1) after glyphosate or sulfoxaflor exposure. Functional annotation using Gene Ontology was performed for the three experiments (Figure 4). It showed that the most affected processes (Figure 4A) after Amistar exposure were carbohydrate metabolic process, lipid transport, and proteolysis. For molecular functions (Figure 4B), the most impacted were lipid transporter activity, chitin binding, protein binding, serine-type endopeptidase inhibitor, ATP binding, and zinc ion binding. For glyphosate, various molecular functions were identified for the DEPs, namely transferase activity, hydrolase activity, molecular function regulator activity, oxidoreductase activity, antioxidant activity, and catalytic activity. The biological processes found were cellular-modified amino acid metabolic process, cell differentiation, anatomical structure development, defence response to other organisms, reproductive process, carbohydrate derivative metabolic process, cell adhesion, establishment or maintenance of cell polarity, immune system process, and metal ion homeostasis. The lists of the identified proteins in each biological process are available in Table S5. Interestingly, from the processes listed above, the protein ATL64812.1 was found to be differentially expressed after both Amistar and glyphosate exposure. This peptidoglycan recognition protein is known to play an important role in the response of insects to bacteria, and according to our OmicsBox interrogation was found to be involved in defence responses to other organisms and immune system processes. This protein was up-regulated after glyphosate exposure (glyphosate + *C. bombi* versus *C. bombi* and glyphosate versus *C. bombi*) and down-regulated after exposure to *C. bombi* alone. For sulfoxaflor, only one biological process, lipid metabolic process, and two molecular functions, hydrolase activity and regulator activity, were identified. 

In this section, we focused on investigating the variation in proteins that could be markers of pesticide and/or pathogen exposure. Specifically, we examined proteins that were shown to play roles, or to be key in immune response, response to stimulus/stress, and response to oxidative stress. Indeed, we observed that following Amistar exposure, nearly all proteins involved in the processes mentioned above were up-regulated. When Amistar was compared to the control, 14 up- versus 3 down-regulated proteins were found. For Amistar + *C. bombi* versus the control, 19 up- versus 14 down-regulated proteins were highlighted, while for the pairwise samples Amistar + *C. bombi* versus *C. bombi*, 16 up- versus 6 down-regulated proteins were identified. Here we suggest that Amistar activates the processes of bee immunity in contrast to *C. bombi*. This was further supported when we compared *C. bombi* to the other conditions, as this time more proteins were down-regulated: *C. bombi* versus control, 6 down and 2 up; and *C. bombi* versus Amistar, 10 down and only one up. Furthermore, some proteins of interest have been shown to be involved in response to stimuli and defence mechanisms [65,66,67,68,69,70]. As examples, chitinase-like protein (XP_016769017.1 and XP_012237228.1), interferon-related developmental regulator 1-like (XP_017879492.1), heat-shock 70 kDa protein cognate 4 (KMQ87979.1), transferrin-like (XP_035740737.1), apolipophorins, and the two proteins ferritin (ABV68875.1) and vitellogenin (AUX13057.1) that were up-regulated only when Amistar was compared to another condition. We also observed that some of these proteins were up-regulated when associated with Amistar and down-regulated when associated with *C. bombi*. In addition, sugar metabolism appeared to be stimulated after bees were exposed to Amistar. Specifically, glucose dehydrogenase (XP_020718843.1) and pyruvate kinase (KYQ58406.1) were up-regulated 54.30 and 100 times in the Amistar + *C. bombi* versus *C. bombi* and Amistar + *C. bombi* versus control comparisons, respectively. However, glucose dehydrogenase was down-regulated when we compared *C. bombi* to Amistar (ratio 0.04). 

A total of 25 DEPs were identified following glyphosate exposure, with 10 being up- and 15 down-regulated. When *C. bombi* was present (alone or in combination with the treatment), almost all DEPs were down-regulated (Table S4). For example, if *C. bombi* was present, the proteins peptidoglycan recognition protein SA (ATL64812.1, ratio 0.08), the uncharacterised protein LOC107189219 (XP_015433190.1), arginine kinase isoform X (XP_039309898.1, ratio 1 0.03), titin-like (LOC100881637), transcript variant X4 (CAB0031481.1, ratio 0.07), and the peroxidase-like isoform X1 (XP_012141527.1, ratio 0.42) were down-regulated. However, arginine kinase isoform X1 (XP_032455210.1), peptidoglycan recognition protein SA (ATL64812.1), and the uncharacterised protein LOC107189219 (XP_015433190.1) were up-regulated when glyphosate was present (alone or combined with *C. bombi*). 

For sulfoxaflor, more than 50% of the DEPs were up-regulated. Following sulfoxaflor exposure and compared to *C. bombi*, up-regulation of proteins involved in defence systems, namely chymotrypsin inhibitor-like (XP_003708656.1, ratio 80.98) and heat-shock protein beta-1 (KYQ52813.1, ratio 95.82), was seen, in addition to up-regulation of an uncharacterised protein LOC107189219 (XP_015433190.1, ratio 50.60). Similar proteins were observed to be up-regulated when we compared sulfoxaflor + *C. bombi* versus *C. bombi*. These proteins were observed to be down-regulated when *C. bombi* was present compared to the control. This seems to be a common response of the bees to the pesticides compared to *C. bombi*, as discussed above. However, no DEPs were detected when sulfoxaflor was compared to the control, even when combined with *C. bombi* (*p* > 0.05). 

**Figure 5:**
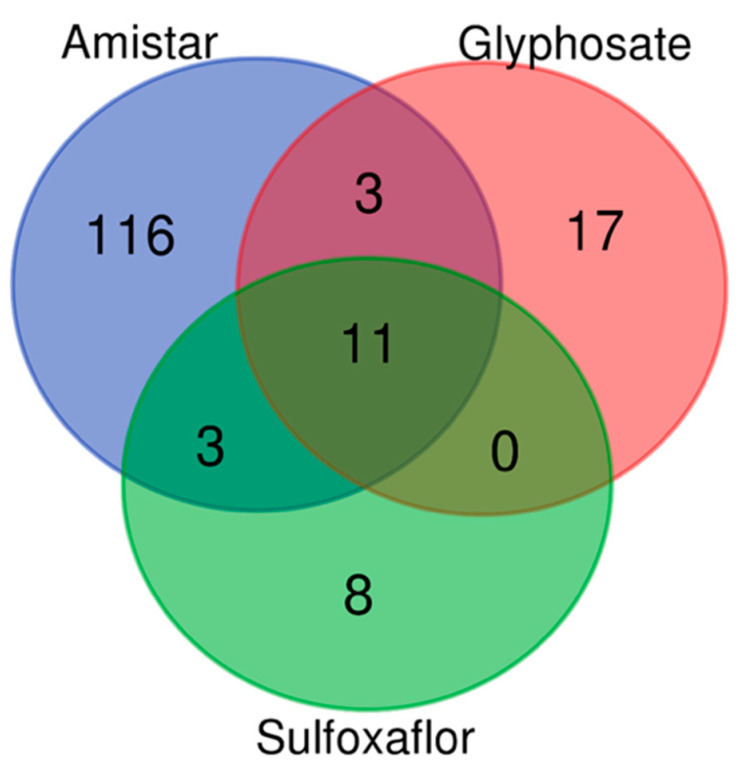
Venn diagram showing the number of common and specific pathways associated with the dysregulated proteins in response to Amistar, glyphosate, and sulfoxaflor.

Furthermore, we examined the most impacted molecular pathways following pesticide exposure. All pathways and proteins are available in Table S6. After Amistar exposure, 133 impacted pathways had at least one DEP involved. In contrast, there were 31 after glyphosate and 22 after sulfoxaflor exposure. We also analysed the overlap between them (Figure 5). The list of these pathways (common and specific) is available in Table S7.

The top 15 most impacted pathways by exposure to Amistar, glyphosate, and sulfoxaflor are illustrated in Table 2. Interestingly, the pathway “Neutrophil degranulation_ R-DME-6798695” (Figure S5), which belongs to the innate immune system, was common to Amistar and glyphosate responses. It is involved in immune responses to bacterial infection [71,72,73]. In our study, the impact on protein abundance (Figure S5, Table S4) depended on the substance. Indeed, we found abundance changed either consistently up or down, or varied, depending on the treatment (Figure S5). The down-regulated proteins when the bees were exposed to glyphosate treatment were transferrin (XP_003486912.1, ratio 0.01 and *p* < 0.01), peroxidase-like isoform X1 (XP_012141527.1, ratio 0.417 and *p* < 0.05), and antichymotrypsin-2-like isoform X4 (XP_033189693.1, ratio 0.018 and *p* < 0.05). However, the protein transferrin (XP_003486912.1) was up-regulated when the bees were exposed to *C. bombi* compared to the control (ratio 100 and *p* < 0.01). After bee exposure to Amistar, the dysregulated proteins involved in neutrophil degranulation were up- and down-regulated depending on the treatment. Indeed, when Amistar was present (alone or combined with *C. bombi*) compared to other conditions (*C. bombi* or control), the highest number of proteins were up-regulated. Among them, the heat-shock 70 kDa protein cognate 4 (KMQ87979.1) was up-regulated following either Amistar treatment alone (ratio 7.12 and *p* < 0.05) or when combined with *C. bombi* (ratio 12.76 and *p* < 0.01) compared to control.

Additionally, we explored the dynamics of the DEPs and pathways and how they could be connected together. Cytoscape networks (Figure S6) illustrated the most important proteins (forming clusters) and their associated pathways that are key in the response to the stressors investigated in this paper. For Amistar, we identified a protein–pathway network with 166 nodes and 207 edges; among them, a cluster was formed with 22 proteins showing the highest number of inter-connexions. For glyphosate, the network consists of 41 nodes and 38 edges, with only 5 connected proteins. Lastly, for sulfoxaflor, we identified fewer dynamics with 26 nodes and 22 edges without connection between the corresponding DEPs. The average number of neighbours was 2.67, 2, and 1.83 for Amistar, glyphosate, and sulfoxaflor, respectively.

## 3. Material and Methods

The experimental work comprises two sections, the experimental treatment and whole organism metrics, undertaken at Royal Holloway University of London, and the haemoproteome work, performed at BioPark (Archamps, France). To cover all three pesticides, two experiments were conducted, one with just Amistar, and one with both glyphosate and sulfoxaflor. 

### 3.1. Bees

Ten *Bombus terrestris audax* colonies were ordered from Agralan Ltd., Swindon, UK, for the glyphosate and sulfoxaflor experiments and three from Koppert Biological Systems, Haverhill, UK, for the azoxystrobin experiments. They were fed ad libitum sucrose and honey-bee-collected pollen from Thorne, Windsor, UK, and Agralan Ltd., Swindon, UK, respectively. All colonies were queenright. All experiments were performed in a temperature-controlled room at 25 °C ± 2 °C and 60% RH ± 10% RH. The room was kept in either darkness or red light so as to minimise stress to the bees. Ten workers per colony were screened for micro-parasites [43], with no infections detected. Only workers were used in the experiment. Bees were not age controlled as we were following an OECD protocol (see below). 

### 3.2. Pesticides

Glyphosate, sulfoxaflor, and dimethoate were sourced as pure active ingredients; (Sigma-Aldrich, St. Louis, MO, USA) CAS-no: 1071-83-6 (Greyhound Chromatography and Allied Chemicals) CAS-no: 946578-00-3, and (Sigma-Aldrich) CAS-no: 60-51-5, respectively. Azoxystrobin is poorly soluble in most solvents viable for bee testing, so the highly stable formulation (Amistar) was used instead. Amistar was purchased online through Agrigem Ltd. (www.agrigem.co.uk, accessed on 2 September 2019) (formulation identifiers are UK MAPP: 18039, Syngenta ID: A12705B). 

### 3.3. Parasites

The details of the parasite exposure are identical to that in the modified ecotoxicological protocol OECD 247 in [52]. Briefly, bees in parasite treatments were orally fed an inoculum of 10,000 *C. bombi* cells, which is known to lead to a field-realistic infection level [74,75]. Infection was validated by dissection after exposure, and only three samples were found to have a failed infection. The infection was allowed to develop for a week, prior to pesticide exposure. 

### 3.4. Exposure

Bees were allocated to treatments so as to ensure an even allocation of bees per colony per treatment. Bees were acutely and orally exposed to the pesticides, adapted from OECD 247 [76]. The exposure methodology is documented in full in [52] under the section marked modified ecotoxicological protocol OECD 247. Briefly, bees were fed the doses detailed in Table 1 and Table 2 in a 40 µL droplet of sucrose after 2–4 h of starvation. Mortality was recorded until haemolymph extraction, 48 h after exposure. The 200 µg dose of glyphosate and azoxystrobin (as 0.8 µL of Amistar) was chosen as the regulatory standard dose for a limit test. The 0.06 µg dose of sulfoxaflor was chosen as a high, but non-lethal, dose so as to simulate a worst-case sublethal acute exposure. Preliminary data from Alberto Linguadoca (pers. comm.) were used to derive the 0.06 µg value. The glyphosate and glyphosate + *C. bombi* whole organism results (survival, weight change, and parasite intensity) are reported and presented in full in [52] without the sulfoxaflor and sulfoxaflor + *C. bombi* results, which are presented here. The proteomic work on the glyphosate and glyphosate + *C. bombi* experiments is presented here only.

### 3.5. Metrics

Survival, weight change, and parasite intensity were all recorded as per Straw and Brown (2021) [52]. For mortality, model assumptions for mixed effects and Cox proportional hazards models were not met, so chi-squared testing was used. Initially a global test was conducted, followed by individual comparisons of each treatment to the control. For the sulfoxaflor and glyphosate experiment, mortality was too low for chi-square testing, so Fisher’s exact tests were used. In treatments with no mortality, no comparison to the control was performed. Weight change and parasite intensity were analysed using mixed effects linear models. The model used was (Metric~Treatment + (1|Colony)). As all dimethoate-exposed bees died within four hours, they were excluded from analyses. The parasite intensity analysis excluded treatments that were not parasite inoculated. The two experiments were analysed separately.

### 3.6. Haemolymph Extraction

At 48 h post-exposure, bees were moved onto ice until docile (52 min ± 20 min). The bees were weighed to allow for a measurement of weight change from the start of the experiment. Haemolymph was collected according to the method established by Arafah et al. (2019) [63] using a specific haemolymph collection kit. Once docile, bees were held in place using plastic tubing, and their abdomen was punctured using a pulled glass capillary (Sutter Instrument Corp, Model P-30, Novato, CA, USA). The glass capillary was inserted dorsally under the second tergum of the abdomen. A 1–5 μL volume of haemolymph was extracted with light suction. Where a sample was cloudy or brown, it was excluded. The collected haemolymph was transferred into a chilled Eppendorf^®^ LoBind Protein microtube (Sigma-Aldrich, St. Louis, MO, USA) pre-coated with PTU and PMSF to prevent melanisation and proteolysis, respectively. The anaesthetised bee was moved into a standard 1.5 mL Eppendorf^®^ tube. Both the bee and sample were stored on ice and moved to a −20 °C freezer regularly. Haemolymph samples were shipped to BioPark on dry ice. 

### 3.7. Batches

The azoxystrobin experiment (Table 3) was run in a single batch, while the combined sulfoxaflor, glyphosate, and *Crithidia bombi* experiment was split over two days as two batches (Table 4). All experimental conditions were matched between batches, with only a day’s stagger separating the batches as part of a 10-day experiment (82% overlap). 

### 3.8. Haemolymph Analyses: Chemicals and Reagents

For sample preparation and analysis, acetonitrile (ACN) and trifluoroacetic acid (TFA), methanol, and ethanol of LC-MS grade quality or higher were obtained from Carlo Erba Reagents (Val de Reuil, France). For MALDI mass spectrometer calibration, two calibration kits Peptide Standard Calibration II and Protein Standard Calibration I from Bruker Daltonics (Bremen, Germany) were used. Ammonium bicarbonate (ABC), 1,4-dithiothreitol (DTT), 4-vinyl-pyridin (4-VP), phenylthiourea as melanisation inhibitor (PTU), phenylmethylsulfonyl fluoride (PMSF) as protease inhibitor, and alpha-cyano-4-hydroxycinnamic acid (4-HCCA) matrix were from Sigma-Aldrich (St. Louis, MO, USA). Trypsin solution was purchased from Promega. RapiGest™ SF was purchased from Waters. Ultrapure water (MilliQ water; Millipore, Billerica, MA, USA) was used.

### 3.9. Haemolymph Preparation for MALDI Molecular Mass Fingerprint (MFP)

To obtain MFPs by MALDI mass spectrometry (MALDI BeeTyping^®^), haemolymph samples were handled according to the protocol published by Arafah et al. (2019) with modifications to optimise sample analysis [63]. Each individual haemolymph sample was analysed with an AutoFlex III Smartbeam^®^ MALDI-TOF mass spectrometer (Bruker Daltonics, Germany). MFPs were acquired following the Bruker BioTyper^®^ recommendations (matrix, method of sample deposition, and detection) with minor adjustments. Briefly, the haemolymph samples were diluted 1:10 in water acidified with 1% TFA. A volume of 1 µL from each diluted sample was spotted on a MALDI MTP 384 polished ground steel plate (Bruker Daltonics), dried under gentle vacuum, and then mixed with 1 μL of 4-HCCA. Following co-crystallisation of the haemolymph spots with the matrix droplet, MALDI MFPs were recorded in a linear positive mode and in an automatic data acquisition using FlexControl software v3.4 (Bruker Daltonics). The samples were manually spotted in triplicate, each of the three spots being read three times. 

### 3.10. MALDI BeeTyping^®^ Acquisition

For MALDI-MS analysis, the following instrument settings were used: 1.5 kV of electric potential difference, dynamic range of detection of 600–18,000 *m/z*, a global attenuator offset of 70% with 200 Hz laser frequency, and 1000 accumulated laser shots per spectrum of haemolymph. The linear detector gain was set at 1.762 kV with a suppression mass gate up to *m/z* 600 to prevent detector saturation by clusters of the 4-HCCA matrix. Calibration of the mass spectrometer was performed using a standard mixture of peptides and proteins (Peptide Standard Calibration II and Protein Standard Calibration I, Bruker Daltonics) to cover the dynamic range selected for analysis. 

### 3.11. Data Processing and Statistical Analyses

MALDI-MS datasets were submitted to ClinProTools™ 2.2 Software (Bruker Daltonics) for post-processing and statistical analyses. Baseline subtraction and spectral smoothing were applied for all the acquired spectra. The total averaged spectra were calculated based on a signal-to-noise ratio equal to 3 for peak-picking and area calculations. The irrelevant spectra that did not pass the required signal intensity and resolution were excluded from the analysis. A post-processing step involving spectral normalisation of all calculated peak areas was performed with ClinProTools™ software prior to the generation of the principal component analysis (PCA). For intensity comparisons, we used Wilcoxon–Kruskal–Wallis tests. To test normality, we used the *p* of the Anderson–Darling test PAD: if close to 1, the data follow a normal distribution; if close to 0, they do not. In the latter case, further analyses used non-parametric tests. The peak lists generated from the software detail the number of ions (peaks) that are significant (PWKW < 0.0083 (0.05/6)) and are discriminant between the pairwise comparisons. The peak lists are also used to analyse the percentage of significant peaks considered in the experimental condition comparisons.

### 3.12. Bottom-Up Proteomics-Based Nano LC-MS/MS

Based on the MFPs profiles, individual bees were selected to form pools for label-free quantitative bottom-up proteomics analyses by liquid chromatography–electrospray ionisation tandem mass spectrometry (LC-ESI-MS/MS). The same control and *C. bombi* pools were used for both the sulfoxaflor and glyphosate batches.

For each experimental condition, three pools composed of five individual haemolymph samples were prepared. The pools were dried by vacuum centrifugation (Labconco, Kansas City, MO) before bottom-up proteomics studies according to Masson et al. (2018) [77] and Houdelet et al. (2021) [22]. Briefly, 20 µL of 0.1% RapiGest in 50 mM ABC buffer was added to the samples. After adding 2 μL of 280 mM DTT (disulfide bond reducing agent), tubes were incubated at 56 °C for 45 min in the dark, centrifuged briefly, and then allowed to cool down. A 4 µL volume of 4-VP (alkylating agent to block cysteine residues) was added, followed by a 30 min incubation in the dark at room temperature. A 2 µL volume of 0.2 µg/µL trypsin solution (Promega) was used for protein digestion. The samples were incubated overnight at 37 °C under gentle agitation. To stop the enzymatic reaction and inactivate RapiGest, samples were acidified by 5 µL of ACN 20–10% TFA and incubated for 45 min at 37 °C. The digested samples were centrifuged for 10 min at 15,000 *g*, and 10 µL of the samples was analysed by LC/ESI-MS/MS using an U3000 nano-HPLC connected to a high-resolution Q-Exactive Orbitrap (all instruments Thermo Scientific). The tryptic digests were separated by reverse-phase chromatography on an Acclaim PepMap 100 C_18_ nanocolumn (75 μm internal diameter, 150 mm length, 3 μm granulometry, and 100 Å porosity; Thermo Fisher Scientific, Bremen, Germany) on-line with a concentration micro-precolumn C_18_ PepMap 100 (300 μm internal diameter, 3 μm granulometry, and 100 Å porosity; Thermo Fisher Scientific). The flow rate was set to 300 nL min^−1^ using a diphasic linear gradient of 0.1% formic acid in water (FA, *v*/*v*) as mobile phase A and ACN with 0.1% FA as mobile phase B. A multistep gradient of 155 min started at 2% B for 6 min, reaching 35% B in 120 min, then from 35% to 70% B in 5 min, followed by a plateau for 5 min. The gradient ended with a return to the initial mobile phase condition (2% B) for 4 min and a column stabilisation for 15 min. NanoLC-MS/MS datasets were acquired in positive-ion and data-dependent modes of analysis. Oxidation of methionine and tryptophan residues was selected for dynamic modification and pyridylethyl on cysteine for static modification. The protein databases used to perform the identifications were downloaded from NCBI and contained sequences from Hymenoptera and the relevant parasites.

### 3.13. Label-Free Quantification (LFQ)

The Proteome Discoverer 2.4 (Thermo Fisher Scientific) was used to perform the label-free quantification. Using a consensus method, the ion-based quantification relied on unique and razor peptides, and the peptide abundance calculation was based on intensity following a normalisation of the datasets made of all the peptides characterised in the LC-MSMS runs. The protein quantification was calculated using the summed abundance with subsequent ANOVA tests. The processing workflow was performed on the retention time frame between 20 min and 135 min, with a precursor mass tolerance value set to 20 ppm and a fragment mass tolerance of 0.5 Da. The minimum trace length value was set to 5, and the maximum retention time shift of isotope pattern was equal to 0.2 min. Proteins with a ratio <0.5 (down-regulation) and >2 (up-regulation) were considered as significant along with *p* < 0.05. A post-hoc test (Bonferroni) was considered in order to compare protein abundance between the experimental conditions.

### 3.14. Functional Annotation: Gene Ontology and Pathways Analysis

For functional annotation of the sequences generated from the LC-ESI-MS/MS analyses, the bioinformatic solution OmicBox software (v2.1.10, functional analysis module Blast2Go https://www.biobam.com, accessed on 10 May 2022) was used. To obtain the most complete annotation labels, the analyses were performed using the four cloud-powered algorithms (Blast, InterProScan, GO Mapping, GO slim). Separate lists of dysregulated proteins of the pairwise comparisons were loaded to investigate the biological pathways and the protein functions following bee exposure to sulfoxaflor, Amistar, or glyphosate alone or combined with *C. bombi*. Combined pathway analysis was performed on the annotated sequences (proteins) joining Reactome and KEGG to identify enriched pathways with expression profiles. Furthermore, protein–protein interaction and pathway networks were constructed using Cytoscape (v3.9.1 https://cytoscape.org/, accessed on 27 May 2022). The network was statistically analysed as an undirected graph.

**Figure 6:**
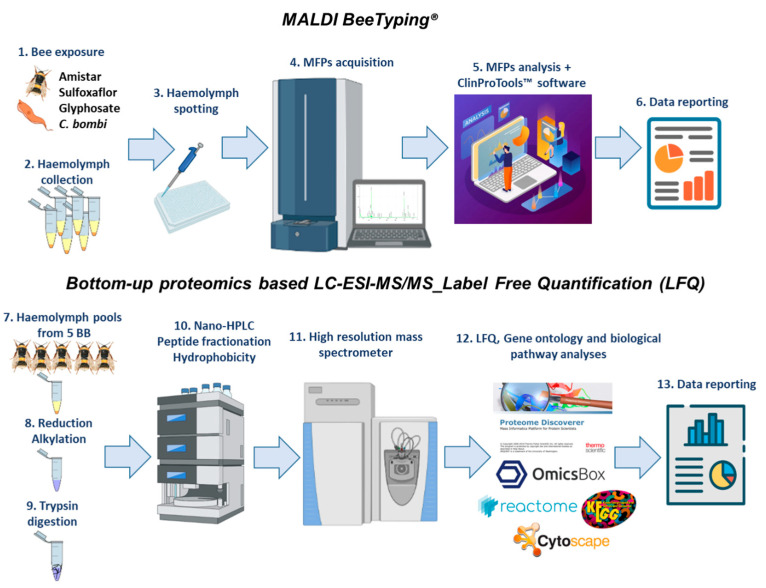
Experimental workflow for exposure, MALDI BeeTyping^®^ (Steps 1–6) and LFQ bottom-up proteomics (Steps 7–13) to follow the impact of stressors on *B. terrestris* health. (**1**) Bee exposure to three different pesticides (Amistar, sulfoxaflor, and glyphosate) and to the parasite *C. bombi*; (**2**) haemolymph collection; (**3**) sample dilution and spotting on MALDI plate; (**4**) MFPs acquisition; data analysis (**5**) and processing (**6**). The Bottom-up proteomics-based liquid chromatography–electrospray ionisation mass spectrometry (LC-ESI-MS/MS): (**7**) pooling of haemolymph from five individual bumble bees 5 (BB); (**8**) chemical reduction/alkylation; (**9**) trypsin digestion; (**10**) fractionation of the digests by nanoliquid chromatography and (**11**) analysis by high-resolution mass spectrometry; (**12**) analysis of the spectra with bioinformatics tools for label-free quantification (LFQ), gene ontology, and biological pathway analyses before (**13**) data reporting.

The complete workflow of the experiments is presented in Figure 6.

## 4. Conclusions

Neither the high dose of glyphosate, nor the sublethal dose of sulfoxaflor caused an observable effect on the whole organism, while the high dose of Amistar caused considerable impacts. However, these whole organism metrics do not capture the totality of the impact of the pesticides, and the haemolymph analysis revealed that, at the doses used in this study, sulfoxaflor has less impact on the *B. terrestris* haemoproteome than glyphosate and Amistar. The latter showed a higher impact across an array of biological processes than either glyphosate or sulfoxaflor. This was observed on the MFPs of individual bees and at the level of the whole haemolymph proteome. However, the trypanosome *C. bombi* showed almost no impact on haemolymph composition. Additional proteomic studies should be carried out on the gut tissue which is the initial target of the parasite *C. bombi*.

## Figures and Tables

**Table 1:** Molecular-related ion variation of the immune peptides apidaecin, abaecin, and chymotrypsin inhibitor across the experimental treatments. The values indicate the percentage of the average peak intensities in one condition versus another one in each pairwise comparison. The value corresponding to the first condition of the comparison is set to 100. Darker green indicates higher levels in the second treatment, whereas lighter green to white indicates lower levels in the second treatment.

Pairwise Comparison	Apidaecin	Abaecin	Chymotrypsin Inhibitor
Control vs. Amistar	277	216	47
Control vs. *C. bombi*	104	NA	134
Amistar vs. *C. bombi*	38	38	280
Control vs. Amistar + *C. bombi*	295	238	29
Amistar vs. Amistar + *C. bombi*	107	110	60
*C. bombi* vs. Amistar + *C. bombi*	283	289	21
Control vs. glyphosate	111	67	123
Control vs. *C. bombi*	108	98	119
Glyphosate vs. *C. bombi*	97	149	97
Control vs. glyphosate + *C. bombi*	125	65	120
Glyphosate vs. glyphosate + *C. bombi*	112	96	98
*C. bombi* vs. glyphosate + *C. bombi*	115	65	101
Control vs. sulfoxaflor	98	116	123
Sulfoxaflor vs. *C. bombi*	111	86	96
Control vs. sulfoxaflor + *C. bombi*	125	90	105
Sulfoxaflor vs. sulfoxaflor + *C. bombi*	128	78	85
*C. bombi* vs. sulfoxaflor + *C. bombi*	116	92	89

**Table 2:** Top 15 most impacted biological pathways and the corresponding number of dysregulated proteins in response to Amistar, sulfoxaflor, and glyphosate.

Condition	Pathway	Protein Number
Amistar	Neutrophil degranulation	13
	Digestion of dietary carbohydrate	5
	Platelet degranulation	5
	Amoebiasis	5
	Common pathway of fibrin clot formation	4
	Glucocorticoid biosynthesis	4
	ECM proteoglycans	4
	COPII-mediated vesicle transport	4
	Cargo concentration in the ER	4
	Regulation of insulin-like growth factor (IGF) transport and uptake by insulin-like growth factor binding proteins (IGFBPs)	4
	Intrinsic pathway of fibrin clot formation	4
	Post-translational protein phosphorylation	4
	mRNA splicing—major pathway	2
	Regulation of insulin-like growth factor (IGF) transport and uptake by insulin-like growth factor binding proteins (IGFBPs)	2
	VLDL assembly	2
Glyphosate	Neutrophil degranulation	3
	Arginine and proline metabolism	3
	Post-translational protein phosphorylation	2
	Platelet degranulation	2
	Regulation of insulin-like growth factor (IGF) transport and uptake by insulin-like growth factor binding proteins (IGFBPs)	2
	Creatine metabolism	1
	Intrinsic pathway of fibrin clot formation	1
	Apoptotic cleavage of cell adhesion proteins	1
	Common pathway of fibrin clot formation	1
	Integrin cell surface interactions	1
	Creatine metabolism	1
	VEGFR2-mediated vascular permeability	1
	Adherens junctions interactions	1
	Glucocorticoid biosynthesis	1
	Antimicrobial peptides	1
Sulfoxaflor	COPII-mediated vesicle transport	1
	Defective C1GALT1C1 causes TNPS	1
	Glycosphingolipid metabolism	1
	Post-translational protein phosphorylation	1
	Platelet degranulation	1
	Intrinsic pathway of fibrin clot formation	1
	Termination of O-glycan biosynthesis	1
	Defective GALNT12 causes CRCS1	1
	Common pathway of fibrin clot formation	1
	Glucocorticoid biosynthesis	1
	Dectin-2 family	1
	Association of TriC/CCT with target proteins during biosynthesis	1
	Cargo concentration in the ER	1
	Regulation of insulin-like growth factor (IGF) transport and uptake by insulin-like growth factor binding proteins (IGFBPs)	1
	Defective GALNT3 causes HFTC	1

**Table 3:** Amistar experiment: Treatment groups, doses of pesticide and parasite given. n = the number of bees that completed the experiment per treatment group, then the number of valid haemolymph samples.

Control (Ctrl) (n = 31, 30)	Amistar Only 200 μg (Equal to 0.8 µL of Pure Amistar) (n = 29, 16)	Dimethoate (Ctrl+)4 μg (n = 29, 0)
*C. bombi* only 10,000 cells (n = 29, 28)	Amistar and *C. bombi* 200 μg (equal to 0.8 µL of pure Amistar) 10,000 cells (n = 32, 26)	

**Table 4:** Glyphosate and sulfoxaflor experiment: Treatment groups, doses of pesticide, and parasite given. n = the number of bees that completed the experiment per treatment group, the number of valid haemolymph samples.

Control (Ctrl) (n = 46, 45)	Glyphosate 200 μg (n = 41, 40)	Sulfoxaflor 0.06 μg (n = 36, 32)	Dimethoate (Ctrl+) 4 μg (n = 36, 0)
*C. bombi* 10,000 cells (n = 36, 31)	Glyphosate and *C. bombi* 200 μg 10,000 cells (n = 35, 35)	Sulfoxaflor and *C. bombi* 0.06 μg 10,000 cells (n = 44, 39)	

## Data Availability

The MALDI-MS datasets have been deposited in the Figshare repository with the doi 10.6084/m9.figshare.21989141. The bottom-up proteomics raw data files and results are available to the readers through the ProteomeXchange Consortium via the PRIDE database identifier PXD039916.

## Supplementary Materials

The following supporting information can be downloaded at: https://www.mdpi.com/article/10.3390/ijms24065384/s1.
