# Supplementary material for: Parasite and Pesticide Impacts on the Bumblebee (Bombus terrestris) Haemolymph Proteome
Source: Int J Mol Sci. 2023 Mar 11;24(6):5384. doi: 10.3390/ijms24065384 (PMC10049270; doi:10.3390/ijms24065384)
Supplement: Supplementary file 1 [file ijms-24-05384-s001.zip › ijms-2234278-supplementary.pdf]

Supplementary materials

Parasite and Pesticide Impacts on the Bumblebee (*Bombus terrestris*) Haemolymph Proteome

Dalel Askri [1,*], Edward A. Straw [2,3], Karim Arafah [1], Sébastien N. Voisin [1,4], Michel Bocquet [5], Mark J. F. Brown [2] and Philippe Bulet [6]

1 Plateforme BioPark d'Archamps, 74160 Archamps, France
2 Centre for Ecology, Evolution & Behaviour, Department of Biological Sciences, School for Life Sciences and the Environment, Royal Holloway University of London, Egham TW20 0EX, UK
3 Department of Botany, School of Natural Sciences, Trinity College Dublin, D02 PN40 Dublin, Ireland
4 Phylogene S.A. 62 RN113, 30620 Bernis, France
5 Apimedia BP22-Pringy, 74371 Annecy, France
6 CR, University Grenoble Alpes, IAB Inserm 1209, CNRS UMR5309, 38000 Grenoble, France
* Correspondence: dalel.askri@biopark-archamps.org

Table S1: The complete lists of MMIs for all pairwise comparisons generated from ClinProTools™ software

Table S2: Immune peptide variation and their corresponding *m*/*z* in all pairwise comparisons

Table S3: All quantified proteins following Amistar, Glyphosate and Sulfoxaflor exposure exported from Proteome Discoverer software

Table S4: Table of DEPs per batch of pesticides (Amistar, Glyphosate and Sulfoxaflor)

Table S5: Biological processes and molecular functions following Amistar, Glyphosate and Sulfoxaflor exposure, details of the corresponding proteins (#seqs)

Table S6: The identified pathways and proteins following Amistar, Glyphosate and Sulfoxaflor exposure

Table S7: The list of common and specific pathways according to each pesticide

All Tables are available in the Excel file: Supp materials_tables_Askri et al IJMS

Figure S1: PCA to discriminate molecular impact on *Bombus terrestris* following Amistar exposure with or without infection with the parasite *Crithidia bombi*

Figure S2: PCA to discriminate molecular impact on *Bombus terrestris* following glyphosate exposure with or without infection with the parasite *C. bombi*.

Figure S3: PCA to discriminate molecular impact on *Bombus terrestris* following sulfoxaflor exposure with or without infection with the parasite *C. bombi*.

Figure S4: Venn Diagram of the specific and common DEPs following Amistar, Glyphosate and Sulfoxaflor exposure

Figure S5: Neutrophil degranulation_R-DME-6798695 pathway and the identified dysregulated proteins following Amistar and glyphosate exposure. The proteins highlighted in green were up-regulated, in red were down-regulated and in orange were observed to be up- and down-regulated depending on the treatment

Figure S6: Cytoscape networks of the DEPs following Amistar, Glyphosate and Sulfoxaflor exposure

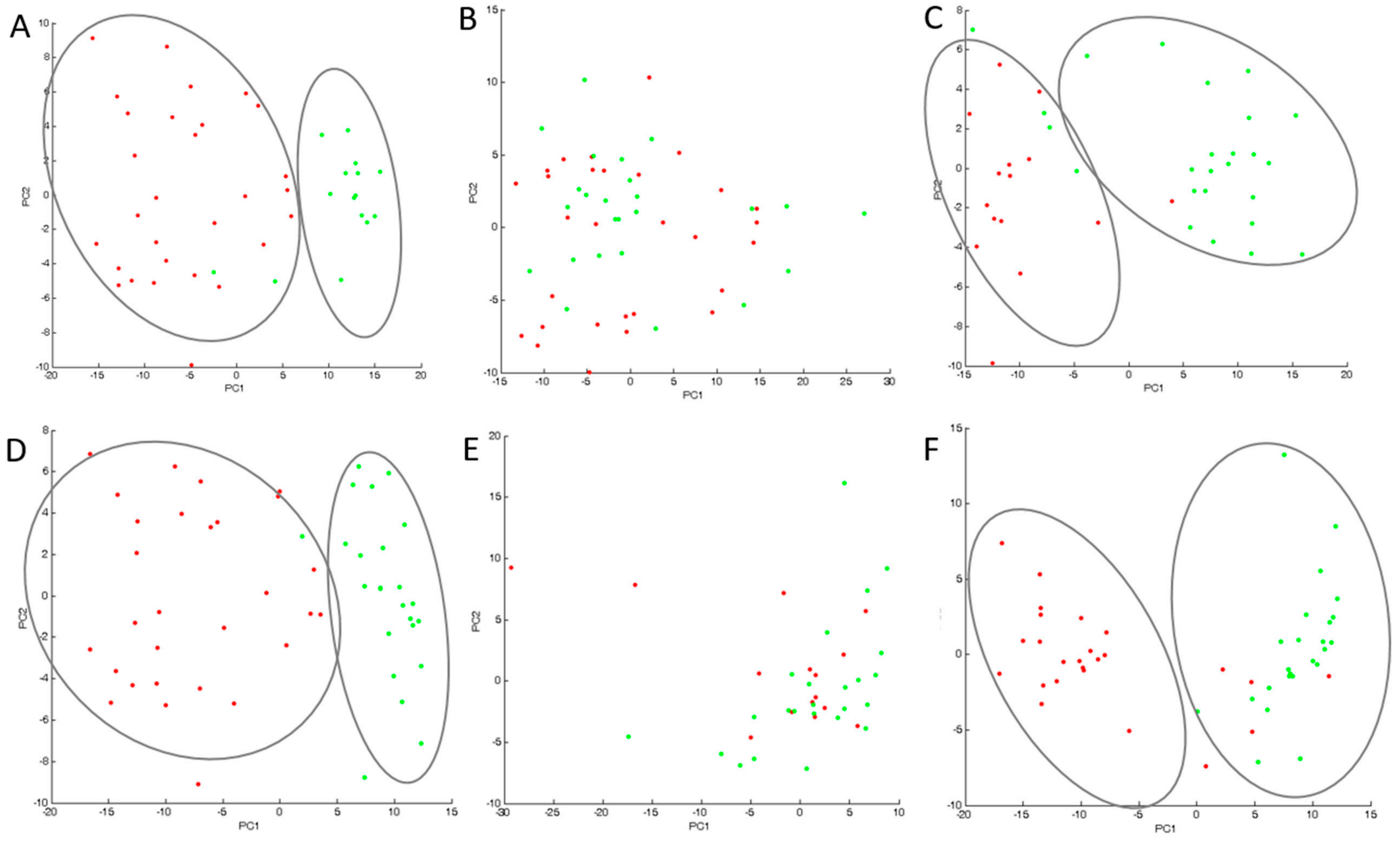

Figure S1: Principal Component Analysis (PCA) to discriminate molecular impact on *Bombus terrestris* following Amistar exposure with or without infection with the parasite *Crithidia bombi*. Control *versus* Amistar (A), control *versus C. bombi*, (B), Amistar *versus C. bombi*, (C), control *versus* Amistar + *C. bombi*, (D),

Amistar *versus* Amistar + *C. bombi*, (E), *C. bombi versus* Amistar + *C. bombi* (F). Each point represents haemolymph molecular mass fingerprints from an individual bee. PCAs are generated from ClinProTools™.

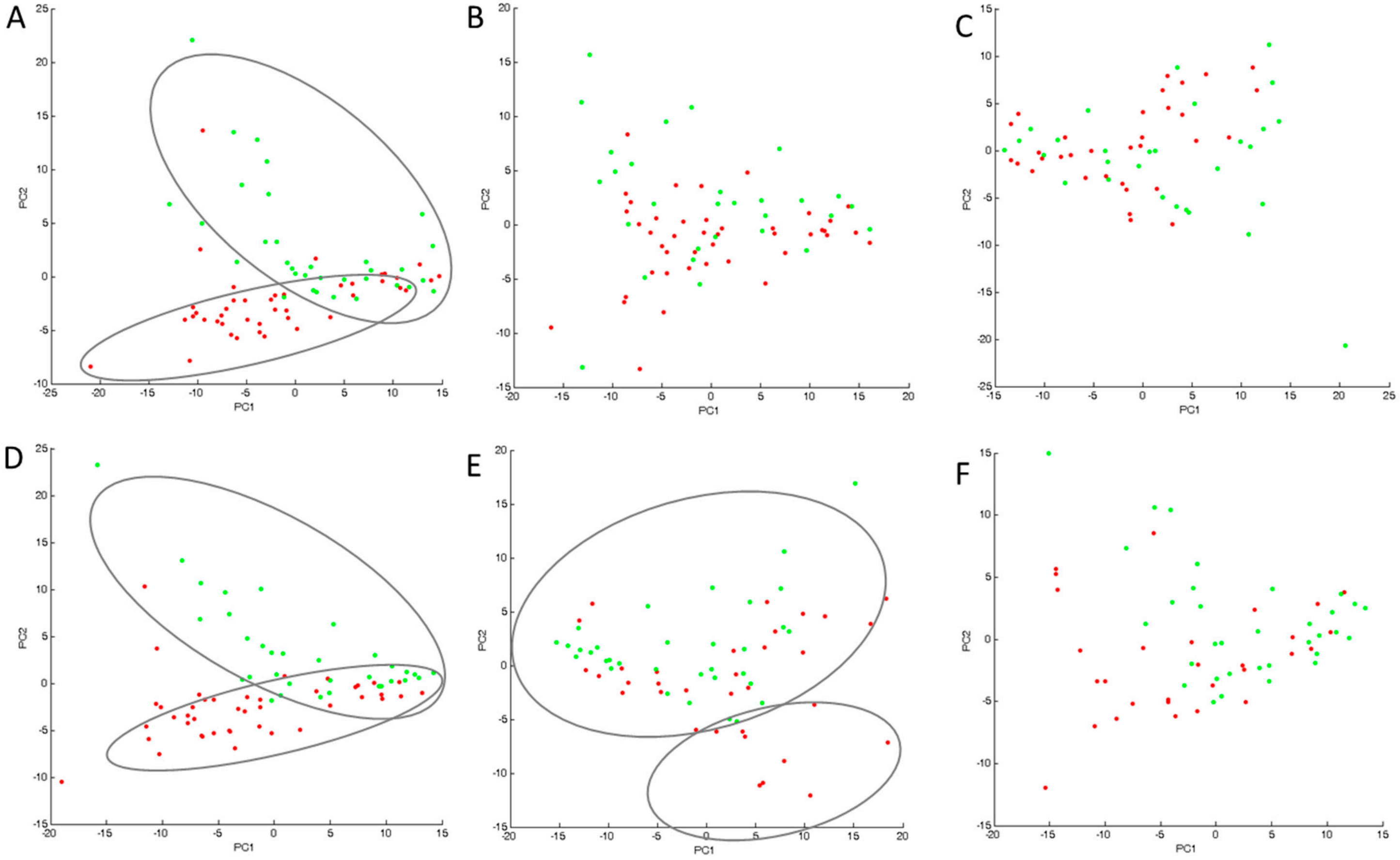

Figure S2: Principal Component Analysis (PCA) to discriminate molecular impact on *Bombus terrestris* following glyphosate exposure with or without infection with the parasite *C. bombi*. Control versus glyphosate (A), control *versus C. bombi* (B), glyphosate *versus C. bombi* (C), control versus glyphosate + *C. bombi* (D),

glyphosate *versus* glyphosate + *C. bombi* (E), *C. bombi versus* glyphosate + *C. bombi* (F). Each point represents haemolymph molecular mass fingerprints from an individual bee. PCAs are generated from ClinProTools™.

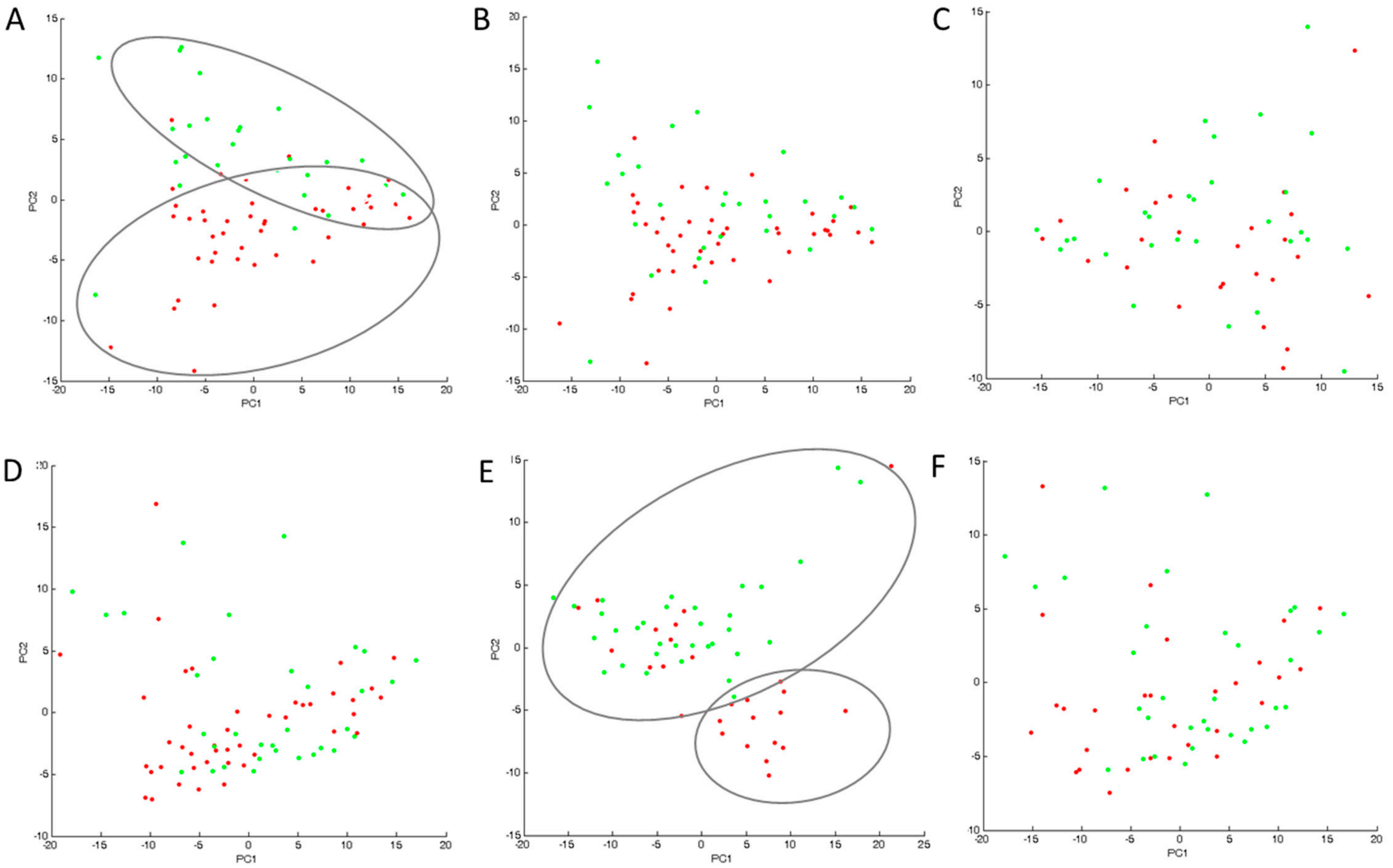

Figure S3: Principal Component Analysis (PCA) to discriminate molecular impact on *Bombus terrestris* following sulfoxaflor exposure with or without infection with the parasite *C. bombi*. Control *versus* sulfoxaflor (A), control versus *C. bombi* (B), sulfoxaflor *versus C. bombi* (C), control versus sulfoxaflor + *C. bombi* (D),

sulfoxaflor *versus* sulfoxaflor + *C. bombi* (E), *C. bombi versus* sulfoxaflor + *C. bombi* (F). Each point represents haemolymph molecular mass fingerprints from an individual bee. PCAs are generated from ClinProTools™.

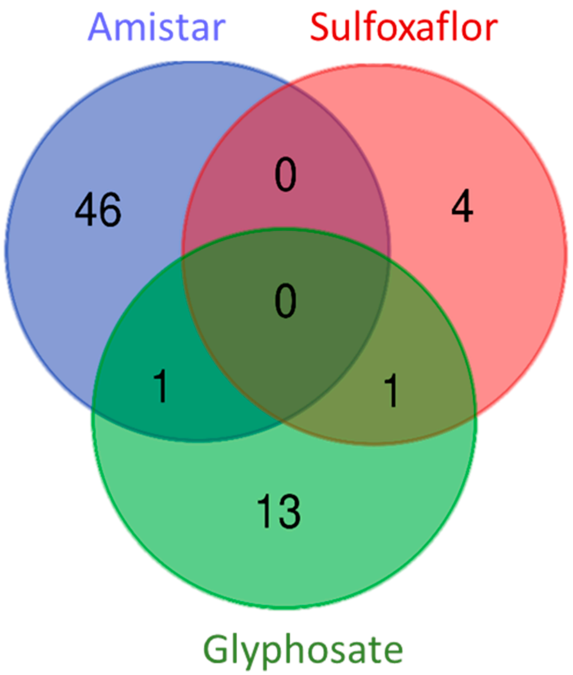

Names	Total	Accession number
Amistar Glyphosate	1	ATL64812.1
Glyphosate Sulfoxaflor	1	XP_015433190.1
Amistar	46	XP_014488121.1 XP_003397944.1 XP_012175644.1 KMQ94768.1 XP_012176635.1 KAF3426068.1 ABV68875.1 XP_012138897.1 XP_020721387.1 XP_029036983.1 A0A2L1U0H7 XP_012172140.1 XP_003398591.1 XP_012169463.2 XP_012148156.1 XP_003397300.2 AUX13057.1 XP_003394859.1 XP_020719356.1 XP_017879492.1 XP_020721219.1 XP_012236328.1 XP_003691494.2 XP_003393186.2 XP_023246016.1 XP_003492760.1 XP_003402576.2 XP_033320019.1 XP_003395541.1 XP_020721343.1 KMQ87979.1 XP_033359707.1 ADG65347.1 XP_014474763.1 XP_012237228.1 XP_012175190.1 XP_012167632.1 XP_020721177.1 XP_033345094.1 XP_035740737.1 XP_016769017.1 XP_003394564.1 KOX76515.1 KYQ58406.1 XP_031833158.1 XP_020718843.1
Sulfoxaflor	4	XP_003708656.1 XP_033196378.1 XP_003398424.1 KYQ52813.1
Glyphosate	13	XP_003486912.1 XP_033357852.1 XP_003403024.1 AEO51760.1 CAB0031481.1 XP_012141527.1 XP_039309898.1 XP_033189693.1 XP_026827010.1 XP_032455210.1 KMQ91288.1 KOX79496.1 XP_003402713.2

Figure S4: Venn diagram showing common responses to chemicals Amistar, sulfoxaflor and glyphosate associated to the corresponding accession numbers of the Differentially Expressed Proteins (DEPs).

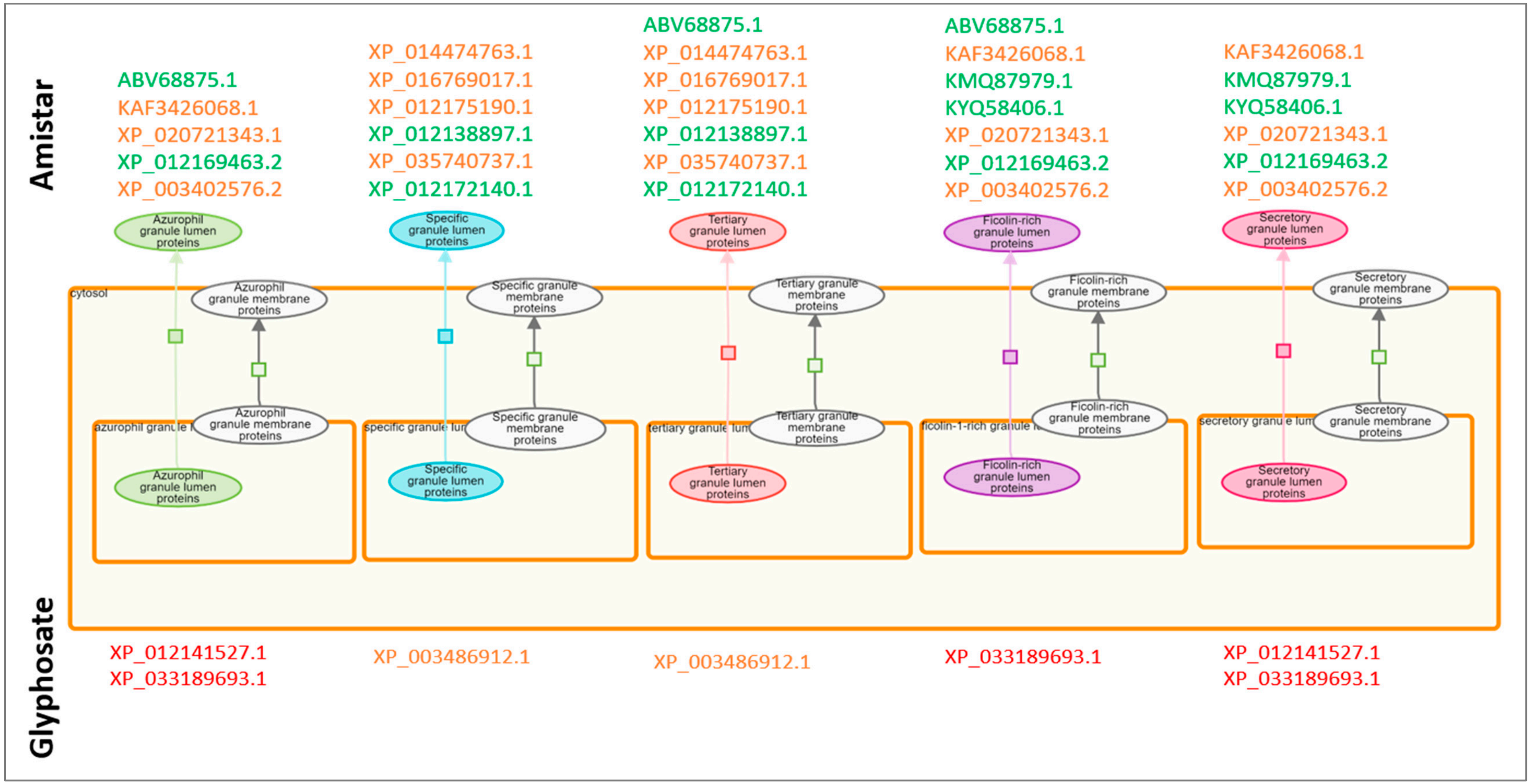

Figure S5: Neutrophil degranulation_R-DME-6798695 pathway and the identified dysregulated proteins following Amistar and glyphosate exposure. The proteins highlighted in green were up-regulated, in red were down-regulated and in orange were observed to be up- and down-regulated depending on the treatment.

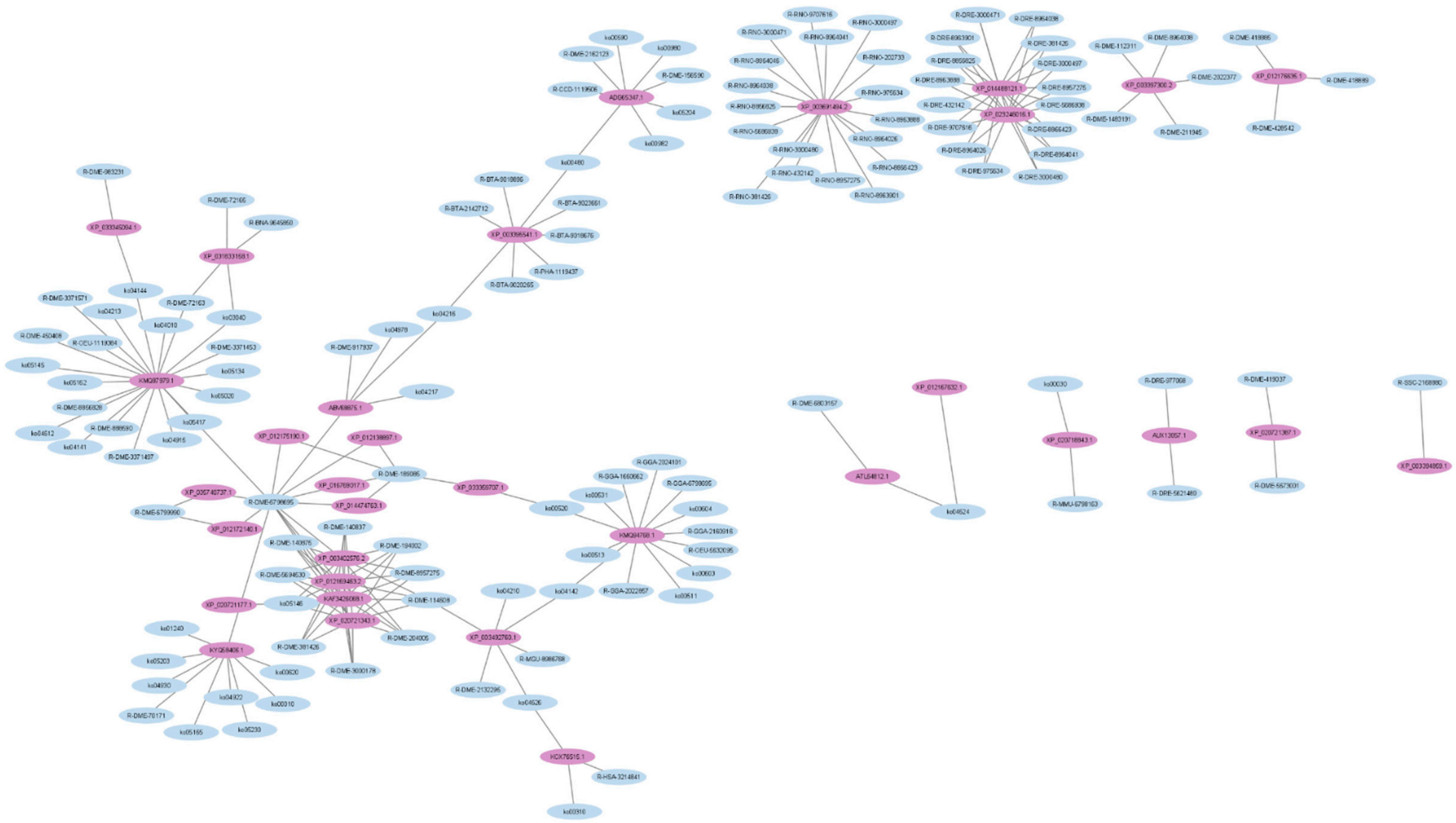

S6. (A) Amistar protein-pathway network with 166 nodes and 207 edges (proteins in purple, pathway identities (IDs in blue), average number of neighbours 2.673.

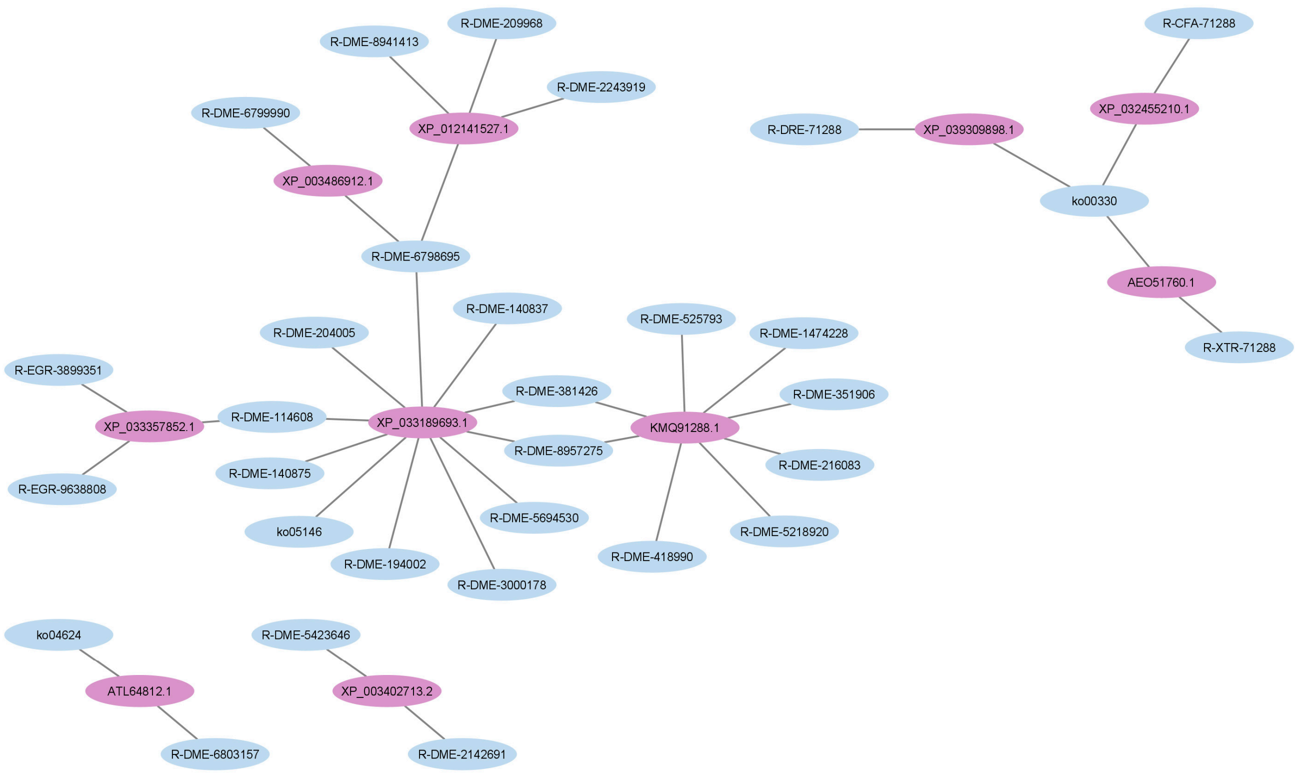

S6. (B) Glyphosate protein-pathway network with 41 nodes and 38 edges (proteins in purple, pathway identities (IDs, in blue), average number of neighbours 2.

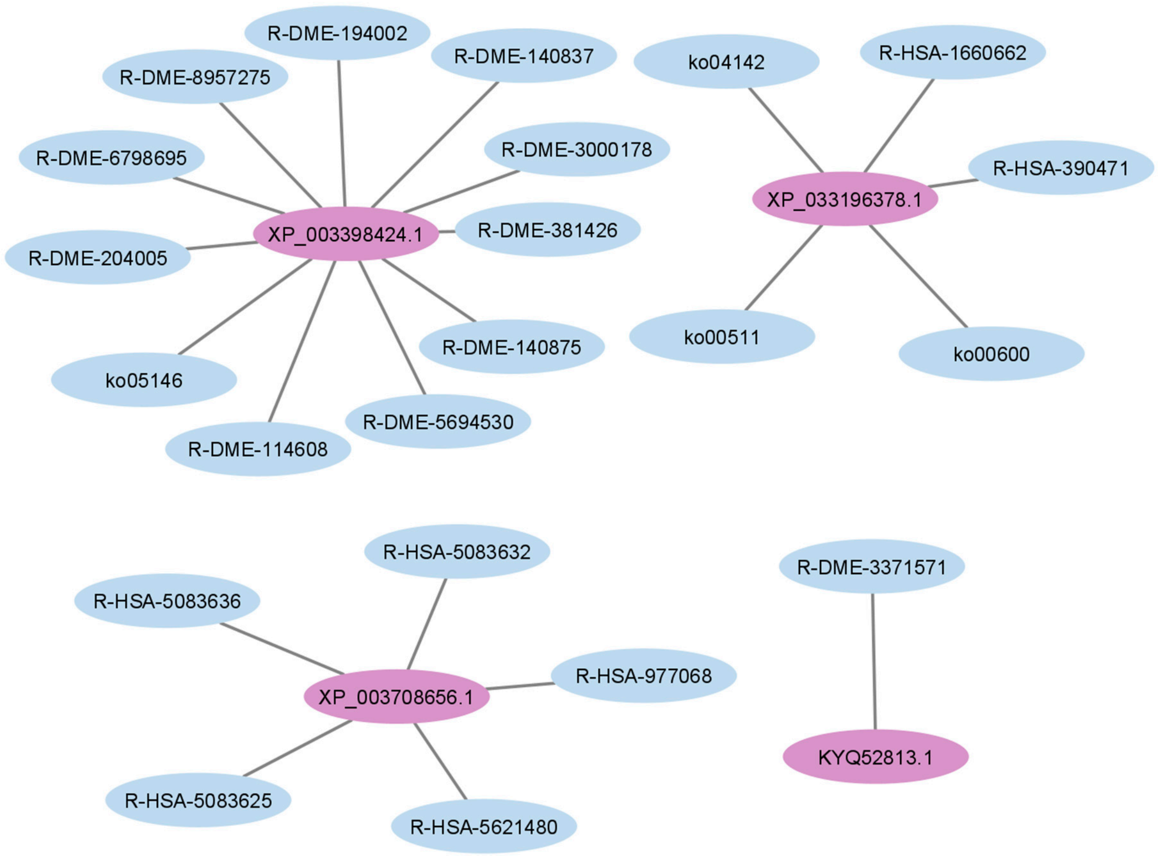

S6. (C) Sulfoxaflor: 26 nodes and 22 edges, average number of neighbours 1.833.

Figure S6: Protein-pathway networks following A) Amistar, B) Glyphosate and C) Sulfoxaflor exposure.

Supplementary Results

Parasite intensity

Amistar Experiment

Bees in the *Crithidia* only treatment had an average parasite intensity of 12,663, while bees in the Amistar + *Crithidia* treatment had an average parasite intensity of 12,281. The difference was not statistically significant relative to the *Crithidia* only treatment (PE = -1,685, CI = -8,967 to 5,946).

Glyphosate and Sulfoxaflor Experiment

Bees in the *Crithidia* only treatment had an average parasite intensity of 20,756, while bees in the Glyphosate + *Crithidia* treatment had an average parasite intensity of 24,124, and those in the Sulfoxaflor + *Crithidia* treatment had an average parasite intensity of 22,605. Neither difference was statistically significant relative to the *Crithidia* only treatment (Glyphosate + *Crithidia*: PE = 3,233, CI = -3620 to 10,062; Sulfoxaflor + *Crithidia*: PE = 1,702, CI = -4970 to 8,350).
